# Supplementary material for: Spontaneous dynamical disordering of borophenes in MgB2 and related metal borides
Source: Nat Commun. 2021 Nov 1;12:6268. doi: 10.1038/s41467-021-26512-4 (PMC8560812; doi:10.1038/s41467-021-26512-4)
Supplement: Supplementary file 1 — Supplementary Information [file 41467_2021_26512_MOESM1_ESM.pdf]

Supporting Information: Spontaneous Dynamical Disordering of Borophenes in MgB_2 and Related Metal Borides

Sichi Li,*[,1] Harini Gunda,[2,3] Keith G. Ray,[1] Chun-Shang Wong,[2] Penghao Xiao,[1] Raymond W. Friddle,[2] Yi-Sheng Liu,[4] ShinYoung Kang,[1] Chaochao Dun,[5] Joshua D. Sugar,[2] Robert D. Kolasinski,[2] Liwen F. Wan,[1] Alexander Baker,[1] Jon Lee,[1] Jeffrey J. Urban,[5] Kabeer Jasuja,[3] Mark D. Allendorf,[2] Vitalie Stavila,*[,2] and Brandon C. Wood*[,1]

1*Materials Science Division, Quantum Simulation Group, Lawrence Livermore National Laboratory, Livermore, CA 94550, United States*

2*Sandia National Laboratories, Livermore, CA 94551, United States*

3*Department of Chemical Engineering, Indian Institute of Technology Gandhinagar, Gujarat 382355, India*

4*The Advanced Light Source, Lawrence Berkeley National Laboratory, Berkeley, CA 94720, United States*

5*Molecular Foundry, Lawrence Berkeley National Laboratory, Berkeley, CA 94720, United States*

E-mail: li77@llnl.gov; vnstavi@sandia.gov; brandonwood@llnl.gov

Supplementary Note 1

To explore boron surface arrangements, we picked a 2x2 boron surface model to start so that a relatively complete configurational enumeration is computational feasible. Our strategy of sampling was to generate a large set of initial configurations so that different potential local minima can be captured. Shown in Supplementary Figure 1, we considered atop and hollow sites relative to the subsurface Mg as the possible locations to place a B atom. Through symmetry determination, placing two B atoms on a 2x2 surface leads to 135 symmetry-distinct initial configurations. The top four alternating B and Mg layers in all initial configurations were then optimized through structural relaxation, while the bottom four layers were frozen staying the same as bulk structure. All local minima found out of 135 structural relaxations were shown in Figure S2.

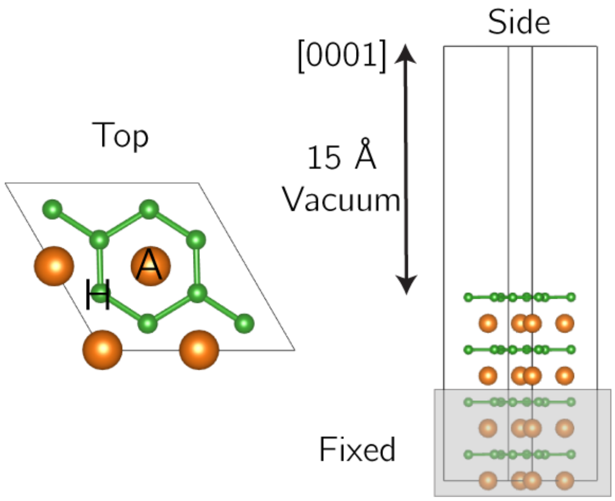

Supplementary Figure 1: Schematic illustration of 2x2 MgB_2 (0001) slab model. A - atop site, H - hollow site, relative to subsurface Mg atoms. Atom color codes: green: B, orange: Mg.

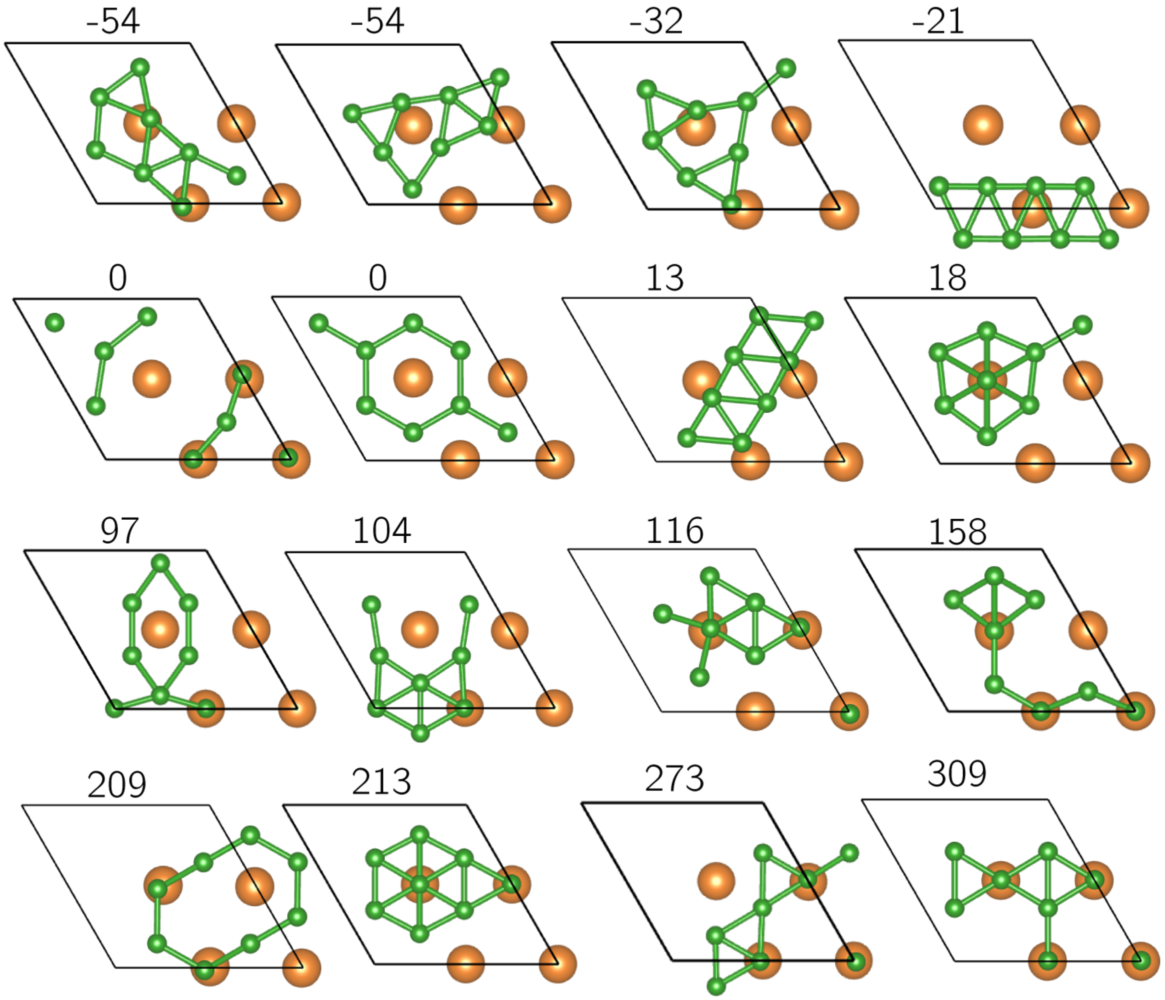

Supplementary Figure 2: Surface structures of all 2x2 MgB_2 (0001) local minima, labeled by DFT-computed energies (meV) per surface boron atoms referenced to the pristine hexagonal surface. Atom color codes: green: B, orange: Mg.

Supplementary Note 2

Supplementary Figure 3 displays the history of structural searching for 4x4 boron surface, by showing structures and DFT-computed energies, referenced to the hexagonal surface and normalized to the number of $B_{surface}$, as a flow diagram of successive ring-opening steps. Starting from the hexagonal surface, in each step we adopted the lowest-energy structure found from the previous step and initiated a ring-opening process by manually moving a pair of neighboring $B_{surface}$ from hollow to atop sites and optimized the structure. For steps with more than one possible neighboring $B_{surface}$ that can be altered, we enumerated symmetry-distinct pairs and tried out all of them. Surprisingly, the first ring-opening leads to a nearly equally-stable surface pattern as the global minimum captured by the 2x2 surface model. The subsequent ring-opening step further stabilizes the surface, and the extent of stabilization depends on the choice of $B_{surface}$ that are altered. The most preferred alternation exposes another two subsurface Mg atoms that are adjacent to the first two exposed Mg at an angle of 120°. For the third ring-opening step, two ways of altering $B_{surface}$ result in further stabilized surfaces while the other two do the opposite. At this point, the lowest-energy surface found contains no visually available $B_{surface}$ pairs to be further distorted. Starting with this structure, we performed Basin-Hopping simulations seeking to further optimize the surface boron layer. After 200 Basin-Hopping steps, we obtained a structure that is 131 meV/$B_{surface}$ lower in energy than the starting hexagonal surface.

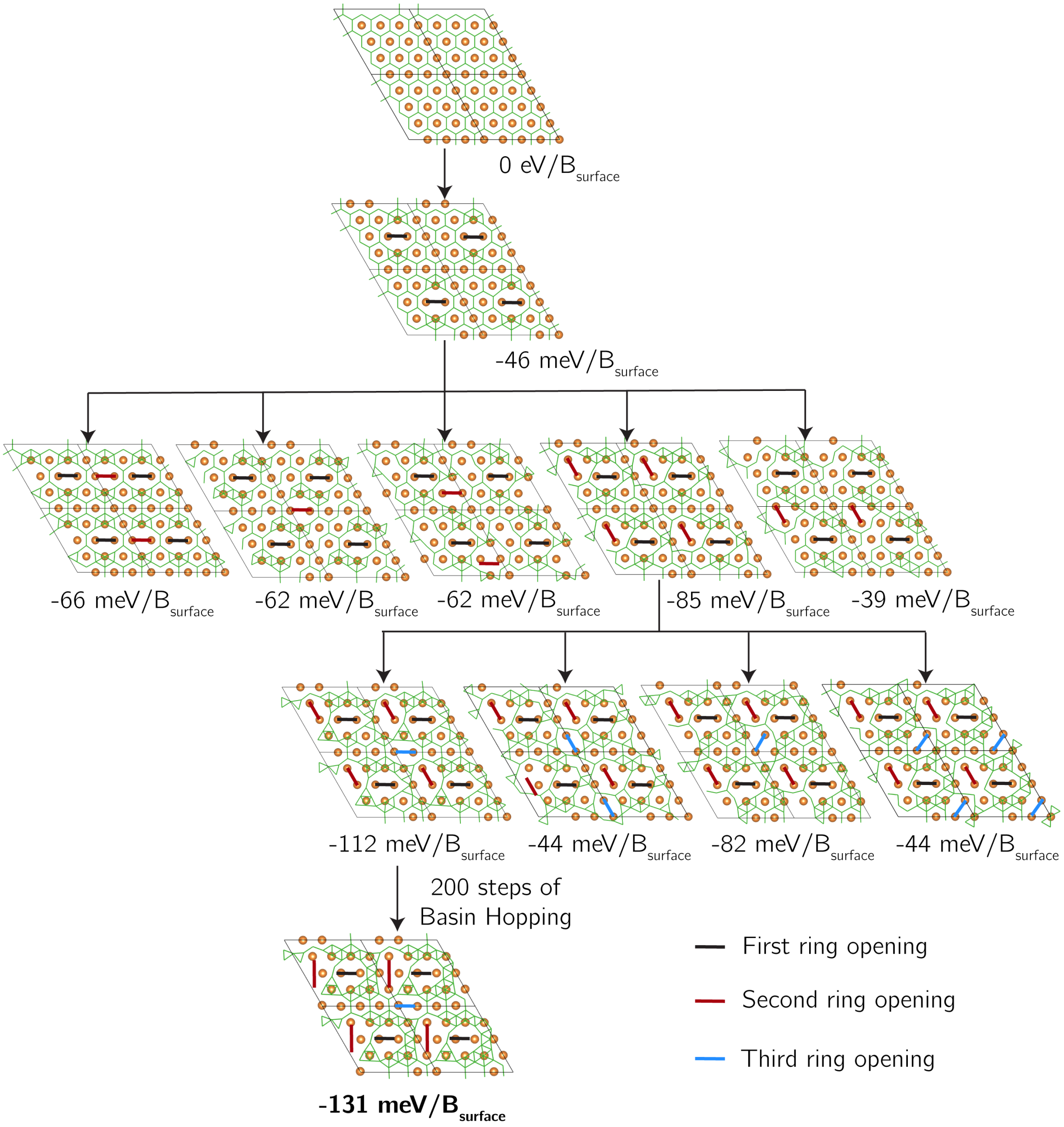

Supplementary Figure 3: Flow diagram of structures and energies per $B_{surface}$, referenced to the pristine hexagonal surface, associated with 4x4 slabs identified in the course of manual+Basin-Hopping structural searching simulations. $B_{surface}$ are displayed as 8x8 surface units for ease of visualization of the local structures around the 4x4 periodic boundary. Lines highlight the ring-opening locations, colored by sequence. Atom color codes: green: B, orange: Mg.

Supplementary Note 3

We performed a series of new calculations to determine the energies of borophenes different in vacancy concentration ($B_{1-x}V_x$) but without constraining the structures to be in crystalline (pristine) state. In addition to 1/3, we included additional 6 vacancy concentration x ranging from 1/12 to 5/12, which can be represented by either 2x2, 3x3 or 4x4 surface models. For 2x2 surface models, we optimized all enumerated symmetry-distinct initial structures. For 3x3 and 4x4 models, full enumeration is again computationally intractable, and we explored local minima by basin-hopping optimizations starting from their pristine crystalline states. Lowest-energy structures for each x were used for subsequent calculations of their formation energies referenced to bulk α-B and clean Mg surface of MgB_2 according to the following equation

$$E^{f}_{B} = (E_{B/Mg\ surface} - E_{Mg\ surface} - N_B E_{\alpha-B})/N_B \tag{1}$$

where $E_{B/Mg\ surface}$, $E_{Mg\ surface}$, $E_{\alpha\text{-}B}$ are DFT-computed energies of B-terminated MgB_2 slab, Mg-terminated MgB_2 slab, and bulk α-B normalized to B atom. N_B is the number of B atoms in the B-terminated MgB_2 surface.

Results including optimized borophene structures and E^{f}_{B} are shown in the Figure S 6. Key findings include: (1) depositing boron to form borophene on Mg surface of MgB_2 using α-B as the boron source is endothermic regardless of boron vacancy concentration x of the resulting borophenes suggesting boron sheets on Mg surface are metastable within the range of computing boron vacancies; (2) borophenes with x close to or larger than 1/3 all exhibit tendency towards disordering; (3) $B_{7/9}V_{2/9}$ is still the lowest-energy borophene polymorph but its energetic difference with the stoichiometric $B_{2/3}V_{1/3}$ is significantly reduced to 0.1 eV/$B_{surface}$ from 0.3 eV/$B_{surface}$ reported by Liu *et al.*[1] where $B_{2/3}V_{1/3}$ is approximated using the pristine hexagonal configuration in their calculations. It is expected that the energetic difference between $B_{7/9}V_{2/9}$ and $B_{2/3}V_{1/3}$ will be further reduced with larger surface

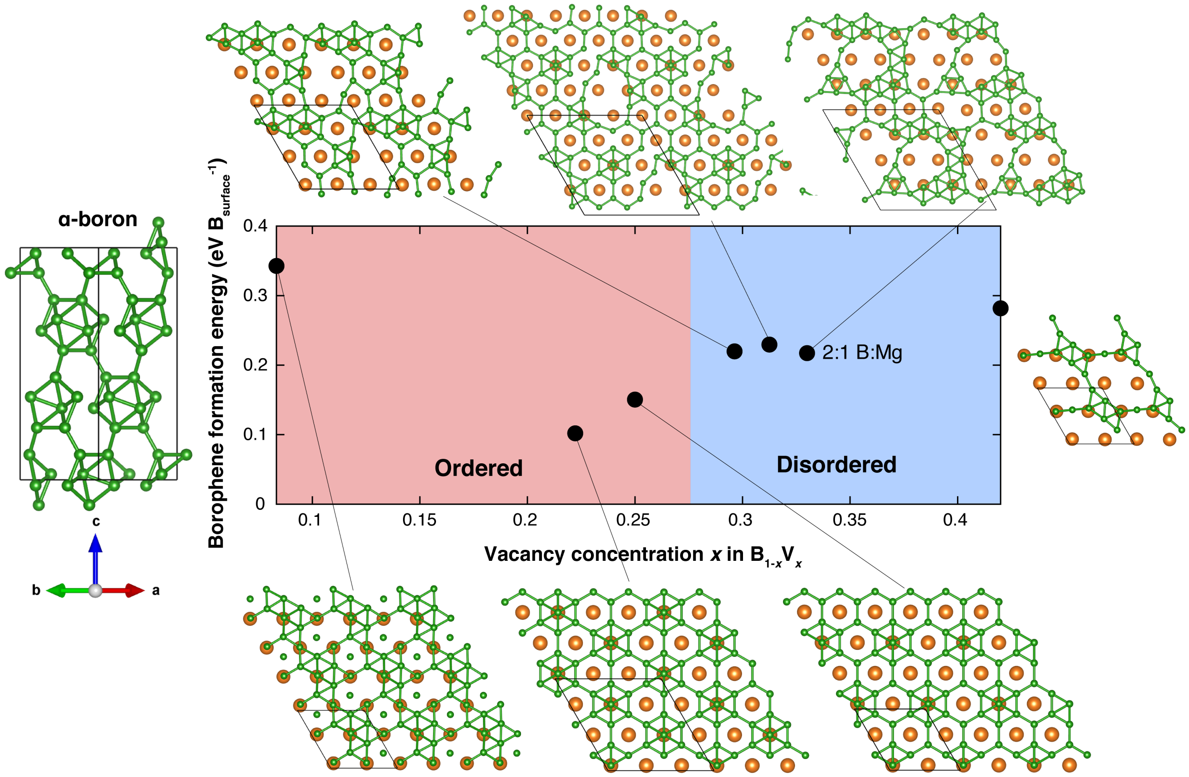

Supplementary Figure 4: DFT-computed structures and E_B^f for boron surfaces with different boron densities.

models which allow higher configurational degree of freedom for geometric relaxation of disordered $B_{2/3}V_{1/3}$. These results suggest that with high boron chemical potential, e.g., under conditions during chemical-vapor deposition, it is likely that boron atoms from boron source will be dispersed on MgB_2 to form the surface pattern lowest in $E_{\mathrm{surface+H}}$ or reach to supersaturated state with even lower boron vacancies as Liu *et al.* proposed. With low boron chemical potential, for example during exfoliation of MgB_2 or under reactive conditions during hydrogenation without additional boron source, global enrichment of surface boron is unlikely although segregation into local B-enriched and boron-deficient regions (where disordering is still preferred) are possible. Furthermore, atomic disordering is expected to introduce excess-entropy to the boron surface that further stabilize the disordered high-vacancy states, analogous to the reported entropic difference (up to 26 J/K mol at 298 K) between crystalline and glass GeO_2 phases.[2]

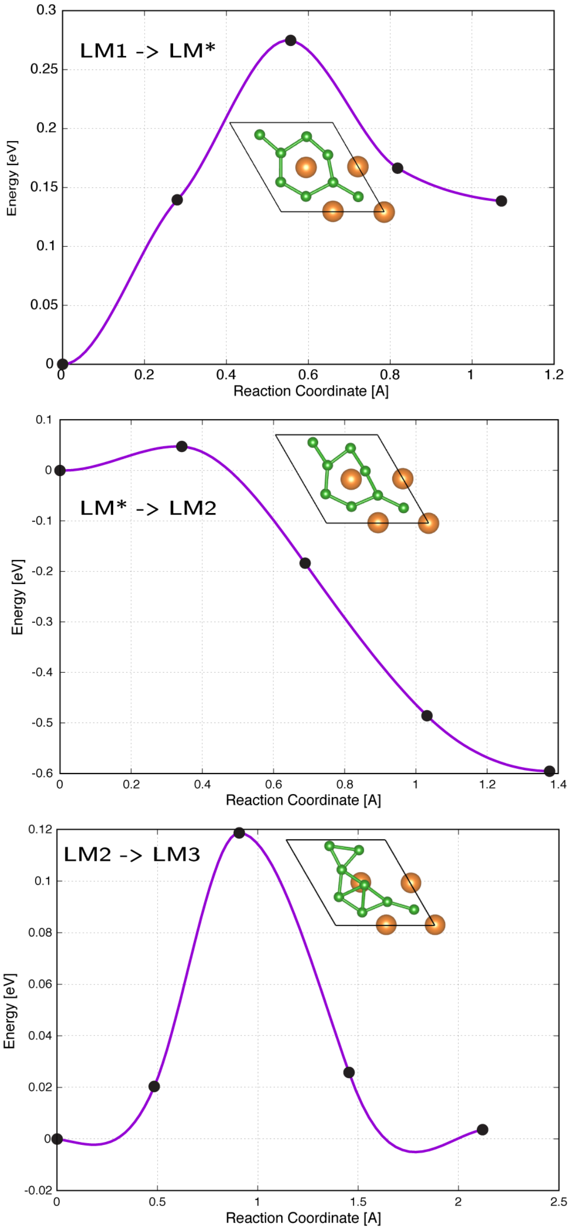

Supplementary Figure 5: Minimum energy paths of LM1→LM*, LM*→LM2, and LM2→LM3 transitions. Transition state structures are superimposed. Atom color codes: green: B, orange: Mg.

Supplementary Note 4

Supplementary Figure 6(a) shows the evolution of CV as a function of metadynamics step. As the purpose of these simulations was to quantify the free energy barrier needed for the surface to break its B-B bonds and reconstruct into other surface patterns, we selected the coordination number of an arbitrarily selected pair of neighboring surface boron atoms as CV. In each step, a Gaussian-shape bias potential with a height of 0.002 eV and width of 0.02 was added to the underlying potential energy surface along the predefine CV. A starting CV value of ≈ 0.8 corresponds to a bonded B-B pair associated with the pristine hexagonal surface (LM1). As potentials were added, the CV first fluctuated with increased amplitude, and then sharply fell to ≈ 0.1, indicating the breaking of the selected B-B bond and reconstruction of the surface. By summing up all potentials added right before the CV fell, we can directly determine the free energy surface at the LM1 local minimum, and the free energy barrier of surface reconstruction.

Supplementary Figure 6(b) shows the free energy surfaces constructed based three replicate simulations all started with LM1 surface pattern but with slightly different starting atomic arrangements. Only small deviations were observed across three replicates, indicating the height and width of Gaussian potentials are small enough so that the constructed free energy surface is well converged without oversampling issues.

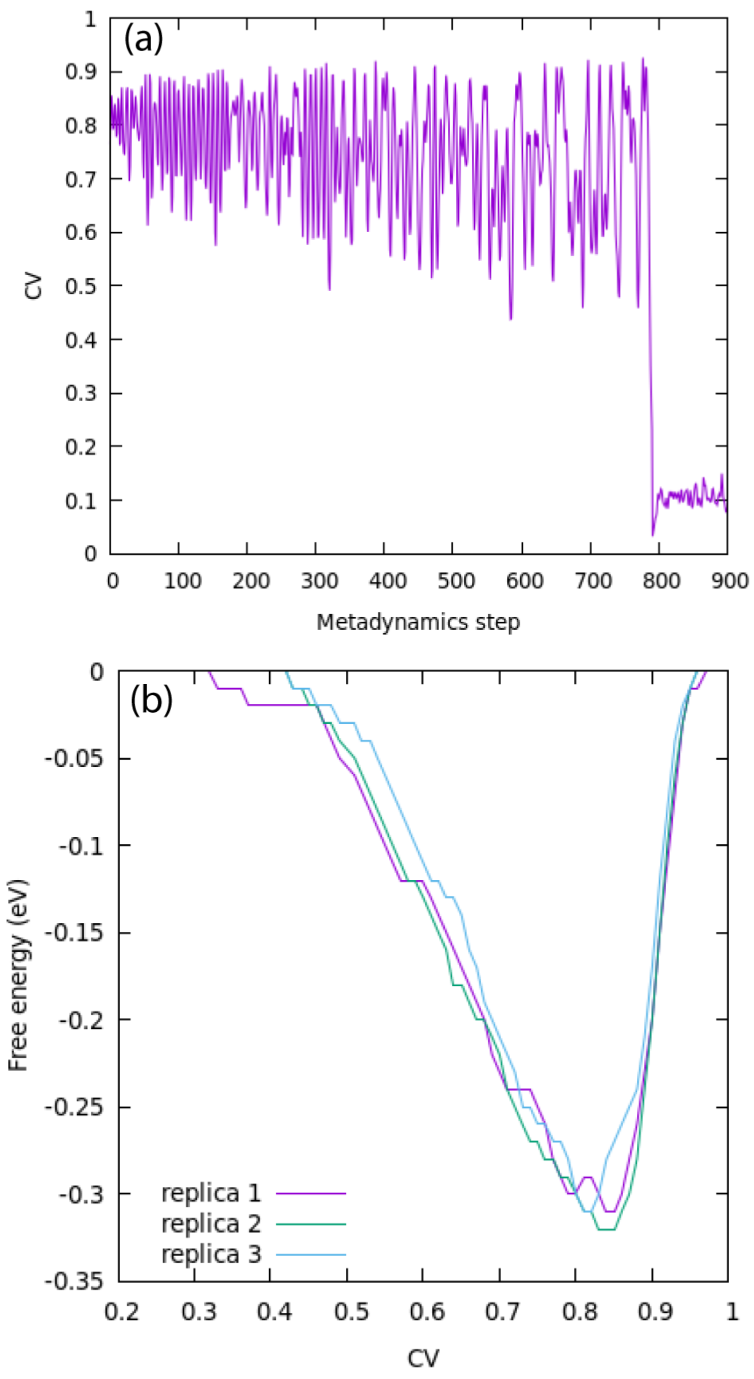

Supplementary Figure 6: (a) CV vs. metadynamics step. (b) Free energy surfaces of LM1 local minimum constructed based on three replicate metadynamics simulations.

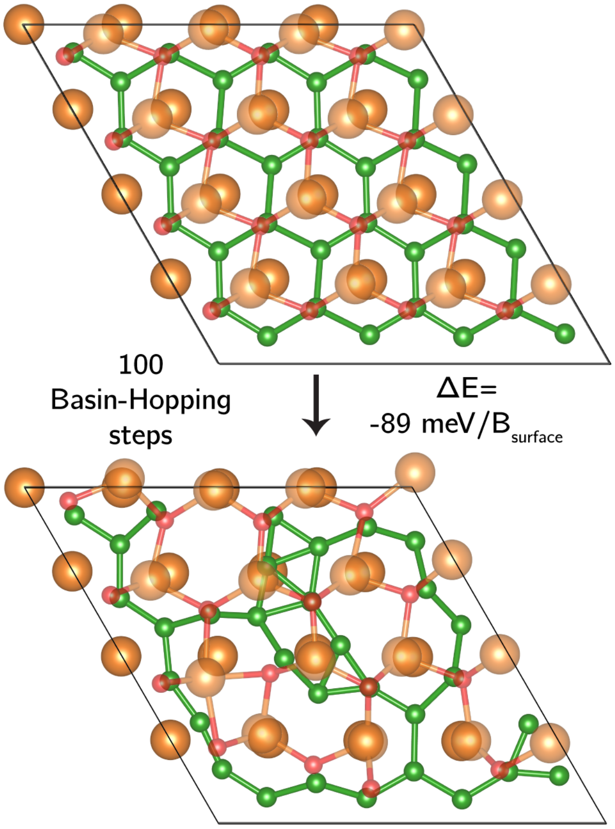

Supplementary Figure 7: The top view of Mg-terminated (0001) surfaces with hexagonal boron subsurface and reconstructed subsurface after Basin-Hopping simulations. Surface Mg+O are rendered with reduced opacity for visualizational purpose. Atom color codes: green: B, orange: Mg. During geometry optimizations, the top four alternating Mg and B layers and the outmost O layer were allowed to relaxed while the bottom four Mg and B layers were fixed. In each Basin-Hopping optimization step, only the coordinates of the top two alternating Mg and B layers and the outmost O layer were randomly distorted.

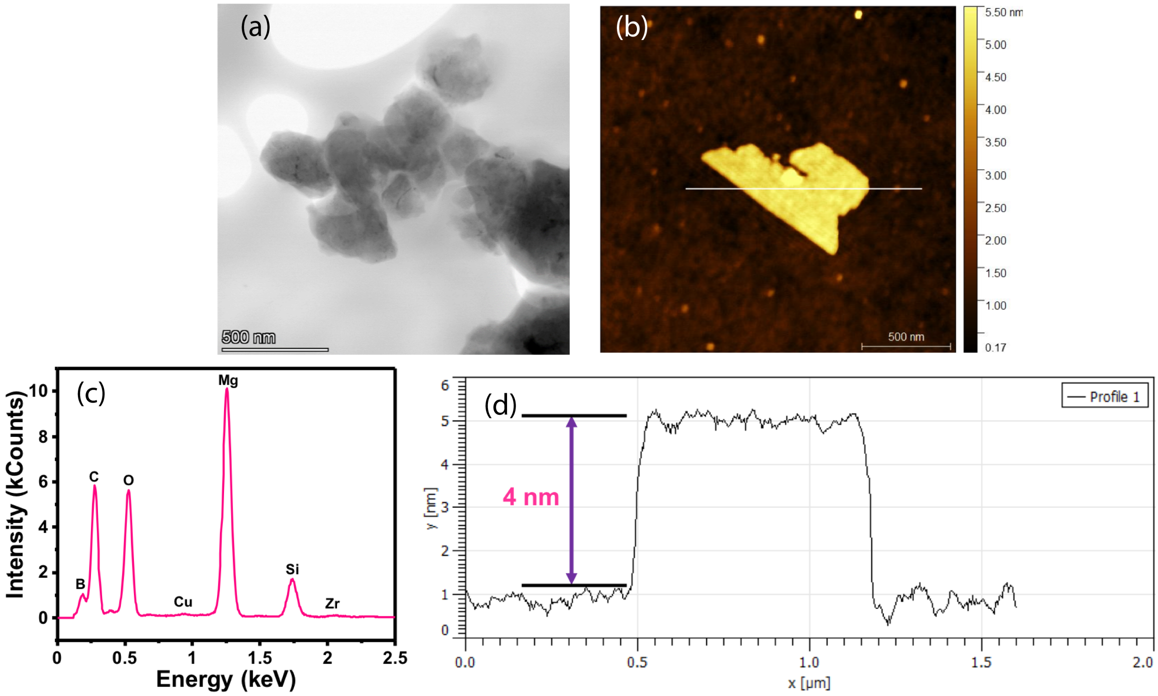

Supplementary Figure 8: (a) Additional TEM image of MgB_2 NSs, (b) Typical AFM image of MgB_2 NSs, (c) EDS analysis, and (d) thickness profile shows that the exfoliated MgB2 nanosheets

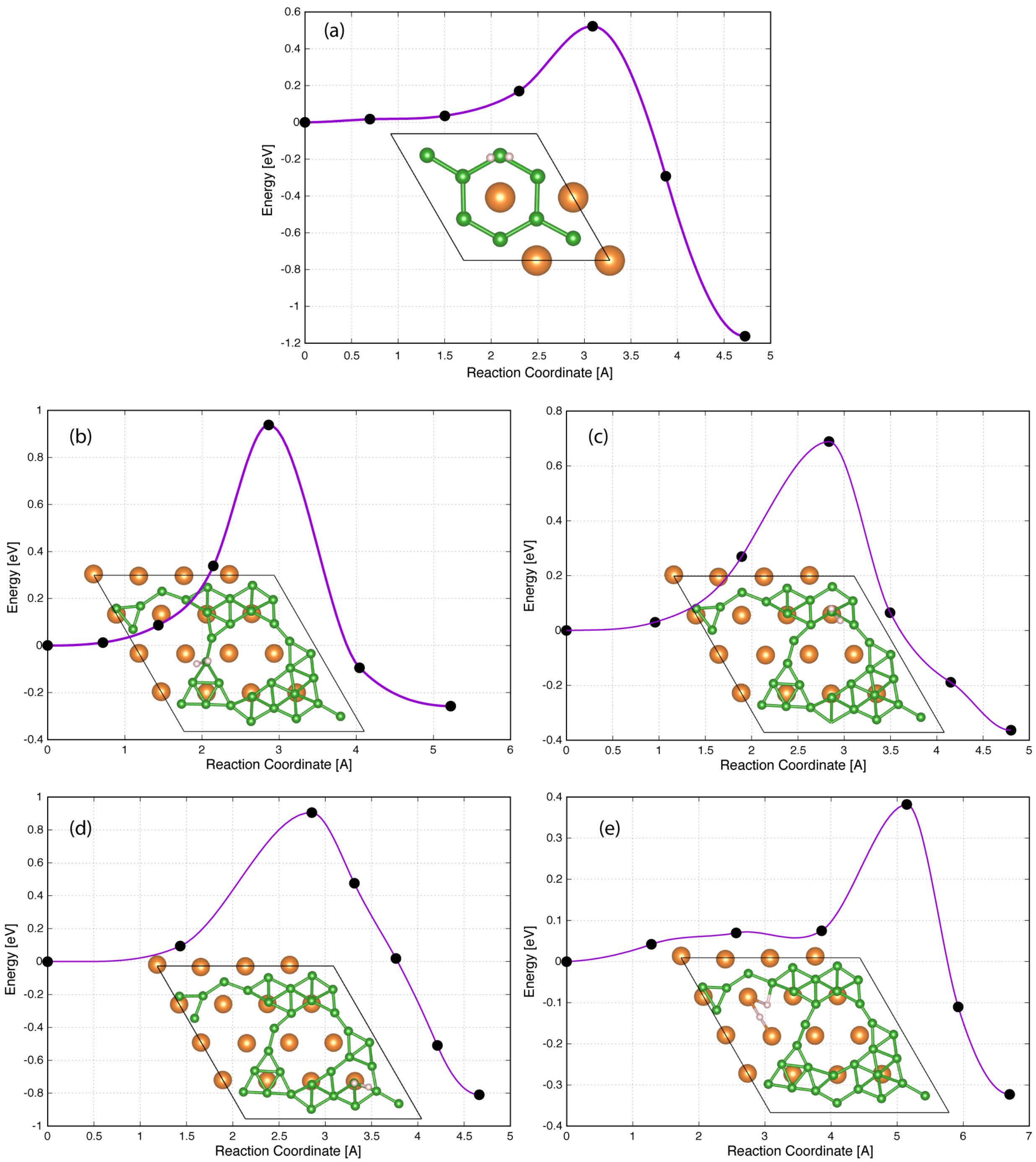

Supplementary Figure 9: Minimum energy paths of H_2 dissociation over (a) the pristine hexagonal surface, (b)-(e) four different pairing sites on the globally optimized surface (LM4). Transition state structures are superimposed. Atom color codes: green: B, orange: Mg, pink: H.

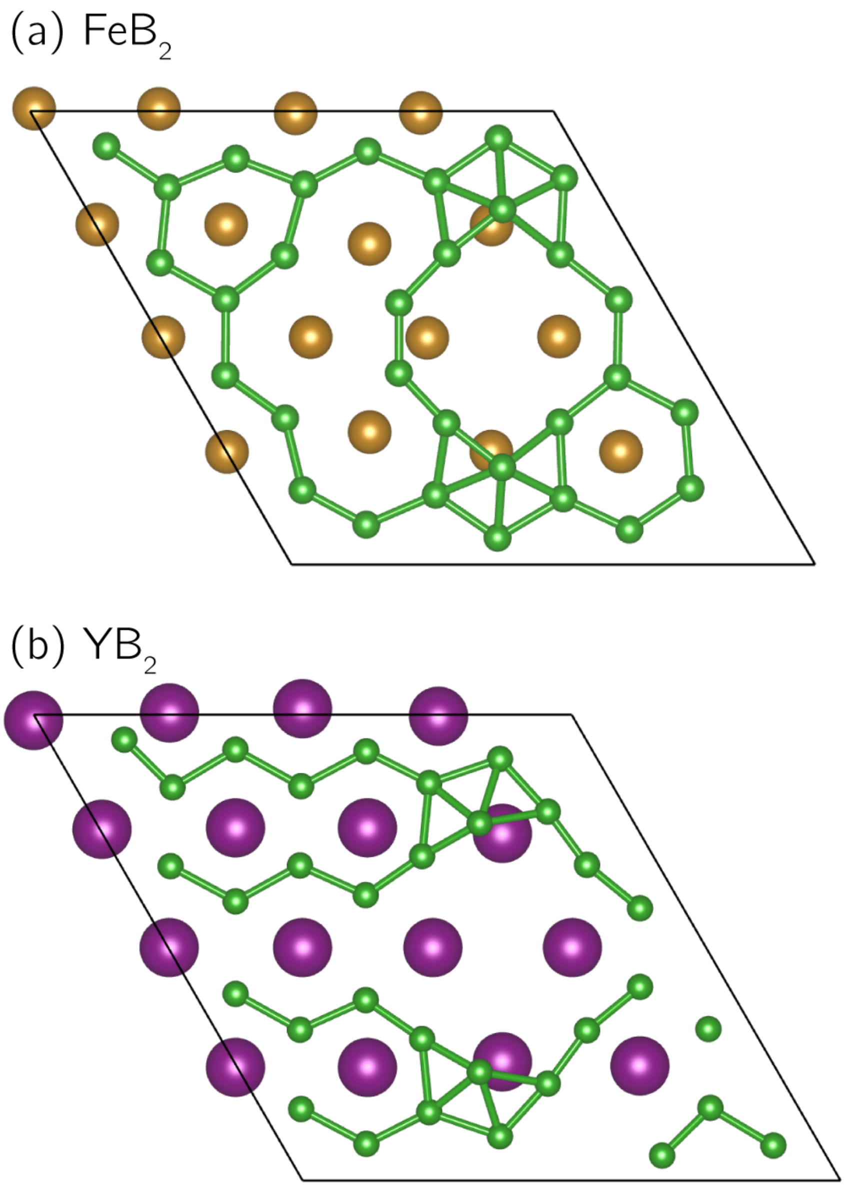

Supplementary Figure 10: Surface structures of FeB_2 and YB_2 after geometry optimization starting with LM* configuration. Atom color codes: green: B, brown: Fe, purple: Y.

Supplementary References

(1) Liu, Y.; Penev, E. S.; Yakobson, B. I. Probing the Synthesis of Two-Dimensional Boron by First-Principles Computations. *Angew. Chem.* **2013**, *52*, 3156–3159

.

(2) Richet, P. GeO2 vs SiO2: Glass transitions and thermodynamic properties of polymorphs. *Physics and Chemistry of Minerals* **1990**, *17*, 79–88

.
